# Longitudinal Study of Salivary Biomarkers in the Definition of Clinico‐Molecular Progression of Parkinson's Disease

**DOI:** 10.1002/acn3.70178

**Published:** 2025-09-27

**Authors:** Maria Ilenia De Bartolo, Daniele Belvisi, Matteo Costanzo, Claudia Caturano, Francesco Emanuele Bellomi, Carolina Cutrona, Flavia Aiello, Giorgio Leodori, Massimo Marano, Romina Mancinelli, Antonella Conte, Giovanni Fabbrini, Alfredo Berardelli, Giorgio Vivacqua

**Affiliations:** ^1^ Department of Radiological Sciences, Oncology and Anatomical Pathology Sapienza University of Rome Rome Italy; ^2^ IRCCS Neuromed Pozzilli Italy; ^3^ Department of Human Neurosciences Sapienza University of Rome Rome Italy; ^4^ Department of Neuroscience Istituto Superiore di Sanità Rome Italy; ^5^ Department of Experimental Morphology and Microscopy, Integrated Research Center (PRAAB) Campus Biomedico University of Rome Rome Italy; ^6^ Department of Neurology and Stroke Unit San Giovanni Addolorata Hospital Rome Italy; ^7^ Unit of Neurology Neurophysiology, Neurobiology and Psychiatry, Department of Medicine, University Campus Bio‐Medico of Rome Rome Italy; ^8^ Fondazione Policlinico Universitario Campus Bio‐Medico Rome Italy; ^9^ Department of Anatomic, Histologic, Forensic Medicine and Locomotor Apparatus Sciences Sapienza University of Roma Rome Italy

**Keywords:** alpha‐synuclein, longitudinal study, neuroinflammation, Parkinson's disease, salivary biomarkers

## Abstract

**Objectives:**

In our previous study, we investigated in de novo PD patients salivary biomarkers targeting different molecular pathways, including alpha‐synuclein (a‐syn), tau pathology, autophagy (MAPLC3beta), and inflammation (TNFalpha). Here, we aimed to investigate longitudinal changes in these salivary biomarkers with the goal of assessing their dynamic changes over time and their predictive value for clinical progression.

**Methods:**

A clinical and molecular 4‐year follow‐up (T1) was conducted on 43 PD patients of our previously molecularly characterized cohort of de novo PD patients (T0). Salivary levels of oligomeric and total a‐syn, pS199‐tau, total‐tau, activated MAP‐LC3beta, and TNFalpha were quantified using ELISA. Clinical assessments included motor and non‐motor symptom scales. The Wilcoxon test was used to verify molecular and clinical variations from T0 to T1; regression analysis to determine whether salivary biomarkers at T0 could predict clinical progression and Spearman's correlations to explore correlations between changes in molecular biomarkers and clinical scores.

**Results:**

Oligomeric a‐syn and MAPLC3beta dramatically decrease, while total a‐syn, phosphorylated‐tau, total‐tau, and TNFalpha exhibited significantly higher levels from T0 to T1. Oligomeric and total a‐syn, phosphorylated and total‐tau at baseline predicted motor progression, while TNFalpha predicted non‐motor worsening. Significant correlations were found for MAPLC3beta, phosphorylated tau, and TNFalpha with motor and non‐motor scores, while no correlations emerged between a‐syn species and clinical scores both at T0 and T1.

**Interpretation:**

Salivary biomarkers dynamically reflect PD progression and predict long‐term clinical outcomes. These findings support the use of saliva as a noninvasive, accessible source for predicting and monitoring disease progression in PD.

## Graphical abstract

Longitudinal changes in salivary biomarkers in Parkinson’s disease (PD) from early (T0) to 4‐year follow‐up (T1), quantified by ELISA: oligomeric and total α‐synuclein, total and phosphorylated tau, MAP1LC3B (autophagy), and TNFa (inflammation). Blue arrows indicate direction of change at T1 vs T0 (up = increase; down = decrease). The right‐hand panel displays each baseline (T0) marker’s relative contribution to predicting motor and non‐motor symptom progression.

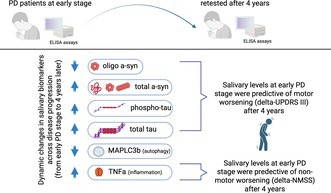

## 1 Introduction

Parkinson's disease (PD) is a progressive neurodegenerative disorder characterized by the accumulation of misfolded α‐synuclein (a‐syn) aggregates and widespread neuronal dysfunction, leading to both motor and non‐motor symptoms [1, 2]. While clinical evaluation remains the cornerstone of PD diagnosis and staging, in recent years several studies have demonstrated that a‐syn detection in peripheral tissue and biological fluids can support clinical diagnosis of PD [3, 4, 5, 6, 7]. Nevertheless, the absence of reliable noninvasive molecular markers for tracking disease progression continues to hamper the development of disease‐modifying strategies. Moreover, although recently proposed PD biological staging systems for PD emphasize the critical importance of defining the disease in molecular terms to inform prognosis and guide the design of personalized treatments [8, 9], many of these approaches rely on invasive sampling or sophisticated techniques that limit their reproducibility and applicability in clinical settings. Furthermore, an additional limitation concerns the current interpretative challenges of Seed Amplification Assays (SAA), which—despite their high sensitivity—may yield negative results even in clinically confirmed synucleinopathies. Factors such as low seed burden, conformational heterogeneity, matrix interference, and pre‐analytical variability can affect assay performance. Furthermore, as recently emphasized by Espay et al. [10], while a positive α‐syn SAA may support a PD diagnosis, it does not inform about disease progression or underlying biological subtype, underscoring the need to complement SAA with quantitative, pathway‐specific biomarkers. In parallel, peripheral biological fluids have gained increasing attention as potential sources of biomarkers reflecting other molecular processes involved in neurodegeneration, such as inflammation, tau pathology, or autophagy [11]. Among these, saliva represents a promising biofluid also for longitudinal molecular monitoring, due to its easy and less invasive collection [7, 12].

The salivary glands and their innervating fibers are directly involved in the early pathological processes of PD, and saliva can mirror hallmark molecular changes, including a‐syn and tau pathology, inflammation, and autophagy dysfunction [12, 13]. In line with this, we have previously demonstrated that different molecular biomarkers—including total and oligomeric a‐syn, total tau, and phosphorylated tau (pS199tau), tumor necrosis factor alpha (TNFa), and the key autophagy protein microtubule‐associated protein light chain 3 beta (MAPLC3b)—can be reliably detected and quantified in the saliva of de novo PD patients, identifying variance in molecular pathways besides a‐syn that could explain the pathological heterogeneity underlying PD subtypes [14]. However, the cross‐sectional design of this study left the predictive and longitudinal behavior of these biomarkers in relation to disease progression unexplored.

In the present study, we performed a 4‐year longitudinal follow‐up of 43 PD patients from the same cohort previously characterized as de novo PD, aiming at (i) assessing the temporal evolution of the salivary biomarkers; (ii) verifying whether their dynamic changes correlate with clinical features and (iii) investigating their predictive value on clinical progression. By integrating clinical data with molecular trajectories, we sought to establish the value of salivary biomarkers as prognostic tools for predicting and monitoring PD progression.

## 2 Materials and Methods

### 2.1 Participants

Patients from a previously characterized cohort of 80 de novo PD patients [14] were re‐evaluated at the Movement Disorders Outpatient Clinic, Department of Human Neuroscience, Sapienza University of Rome, for clinical and salivary follow‐up after a median interval of 4 ± 0.6 years. Among the previous cohort of 80 de novo PD patients, 43 underwent follow‐up. Inclusion criteria for follow‐up required a confirmation of PD diagnosis by a movement disorder specialist, according to the established international clinical criteria [15]. Exclusion criteria included the conversion into a different neurodegenerative disorder at the follow‐up evaluation. Forty age‐ and sex‐matched healthy controls (HC) were recruited among non‐consanguineous relatives of PD patients. Exclusion criteria for HC were neurological or psychiatric disorders or assumption of any drugs known to induce parkinsonism. Clinical assessment of patients comprised disease duration, disease stage as measured by the Hoehn and Yahr (H&Y) scale, disease severity evaluated using the Movement Disorder Society‐sponsored revision of the Unified Parkinson's Disease Rating Scale (MDS‐UPDRS), cognitive performance assessed by the Montreal Cognitive Assessment (MoCA), and non‐motor symptom burden assessed by the Non‐Motor Symptoms Scale (NMSS). Patient re‐enrolment, saliva collection, preprocessing, and storage were carried out at the Department of Human Neurosciences, Sapienza University of Rome, whereas salivary sample analysis was performed at the Laboratory of Microscopic and Ultrastructural Anatomy, Campus Bio‐Medico University of Rome. The initial study was conducted between November 2017 and November 2019 [14], and the current follow‐up study took place from December 2019 to December 2024. The study was approved by the Institutional Ethics Committee, and all participants provided written informed consent prior to enrolment. The study was conducted in accordance with the latest revision of the Declaration of Helsinki.

### 2.2 Saliva Sample Collection and Storage

Sample collection was performed in accordance with previous studies [13, 14, 16]. We collected a minimum amount of 1 mL of saliva from each subject. At the time of collection, the subjects remained for 60 min, without smoking in the previous 4 h and without drinking alcohol in the previous 12 h. Saliva was collected by drooling into a 50 mL vial, which was immediately placed on ice to block proteolytic activity. The samples were then placed in 10 mL Falcon tubes and centrifuged for 20 min at 5000 *g* at 4°C to remove residual particles. After centrifugation, the supernatant was transferred to new 10 mL Falcon‐type tubes and each sample was treated with protease inhibitor cocktail (Sigma Aldrich, USA), at a concentration of 100 μL per 1 mL of saliva. Each sample was then aliquoted into 1 mL Heppendorf‐type tubes and stored at −80°C before biological investigations. Sample storage was performed at −80°C in accordance with previous studies suggesting the stability of different a‐syn forms when stored at this temperature [17, 18].

### 2.3 ELISA Analysis of Samples

Biomarker detection in salivary samples was performed using commercial ELISA kits, following manufacturer protocols adapted for saliva, as established in our previous study [14]. The Anti‐Alpha‐Synuclein Quantitative ELISA kit (SensoLyte 55550) was used to evaluate total a‐syn, whereas the Human A‐Syn Competitive Oligomer ELISA kit (MyBioSource, MBS730762) was used to assess oligomeric a‐syn levels. Salivary levels of t‐tau and pS199tau were determined using the Invitrogen Total Tau ELISA kit (KB0041) and the Invitrogen Human Tau [pS199] ELISA kit (KHB7041), respectively. Estimation of salivary TNFa was performed using the Cloud‐Clone Human TNFalpha ELISA kit (SEA133Hu). Regarding MAPLC3beta, we used the Autophagy ELISA kit (MAP‐LC3beta Quantitative‐ MBS169564) and we adapted the manufacturer's protocol to detect activated MAP‐LC3beta in saliva as we previously described [14]. The concentrations of salivary total a‐syn, a‐syn oligomers, total tau, pS199tau, activated MAP‐LC3b, and TNFa were determined by spectrometric measurement at 450 nm in an appropriate microplate reader (LT 4000, Labtech, Health field, UK). Standard curves were prepared by plotting absorbance readings or optical density values of standards against their concentrations. For total tau, pS199tau, activated MAP‐LC3b, and TNFa, optimization of the standard curves was performed based on the salivary concentrations of these proteins detected in previous studies [19, 20, 21, 22].

### 2.4 Statistical Analysis

Statistical analyses were performed using GraphPad Prism (version 9) and IBM SPSS Statistics (version 30). The Shapiro–Wilk test was applied to assess the normality of all variables. Sex distribution differences between groups were assessed using the chi‐square test. The Mann–Whitney *U* test was used to evaluate differences in clinical and biomarker variables at T0 between patients who dropped out and those who completed follow‐up, as well as to re‐assess differences in salivary biomarkers at T0 between the PD subgroup completing follow‐up and HC. The Wilcoxon signed‐rank test was employed to investigate longitudinal changes in clinical scores and salivary biomarker levels between T0 and T1 time points in PD patients. Spearman's rank correlation coefficients were used to examine associations between salivary and clinical variables at both T0 and T1. *Z*‐scores were calculated for linear regression analyses, with salivary biomarker levels at T0 as predictor variables and the delta of clinical scores as dependent variables (delta score = [T1 score − T0 score]/T0 score). The structure of the regression models was: delta_clinical score = *β*_0_ + *β*_1_·*Z*(total a‐syn) + *β*_2_·*Z*(oligo a‐syn) + *β*_3_·*Z*(total tau) + *β*_4_·*Z*(pS199tau) + *β*_5_·*Z*(MAPLC3b) + *β*_6_·*Z*(TNFa) + *ε*. ROC curve analyses for oligomeric and total α‐synuclein were conducted at T1 to evaluate their accuracy in distinguishing PD patients from HC.

## 3 Results

### 3.1 Comparison of Molecular and Clinical Data at T0 Between Patients Who Did the Follow‐Up and Those Who Did Not

Forty‐three out of 80 patients of the previously studied cohort of de novo PD patients completed clinical and salivary follow‐up. At baseline, no significant differences were observed in age, sex, disease duration, motor and non‐motor symptoms, or salivary biomarker levels between patients who dropped out and those who completed the 4‐year follow‐up. The only significant difference was found in the MoCA score, which was higher in patients who completed follow‐up (median 28, IQR 27–28) compared to those who did not (median 26, IQR 25–29) (*U* = 808.5, *p* = 0.020).

### 3.2 Comparison of Molecular Data at T0 Between Followed‐Up PD Patients and HC

The analysis of the PD patients who completed the follow‐up compared to HC confirmed significant differences in salivary biomarkers at T0. In particular, oligomeric a‐syn levels were significantly higher in PD patients (median = 1.27 ng/mL, IQR: 1.09–2.50) compared to HC (median = 0.14 ng/mL, IQR: 0.11–0.25) (*U* = 1340, *p* < 0.001). Similarly, MAPLC3b concentrations were significantly elevated in PD patients (median = 1.60 pg/mL, IQR: 1.39–2.20) versus HC (median = 0.33 pg/mL, IQR: 0.30–0.37) (*U* = 1256, *p* < 0.001), as were TNFa levels (PD: median = 1.04 pg/mL, IQR: 0.46–2.24; HC: median = 0.63 pg/mL, IQR: 0.54–0.75) (*U* = 888.0, *p* = 0.014). Total tau concentrations were also significantly higher in PD patients (median = 7.86 pg/mL, IQR: 7.41–15.62) compared to HC (median = 3.43 pg/mL, IQR: 2.87–6.17) (*U* = 1241, *p* < 0.001). As expected, no significant differences were found in salivary total a‐syn (*U* = 816.0, *p* = 0.05) and pS199tau (*U* = 149, *p* = 0.180) levels between patients and HC.

### 3.3 Changes of Salivary Molecular Biomarkers From T0 to T1

**FIGURE 1:**
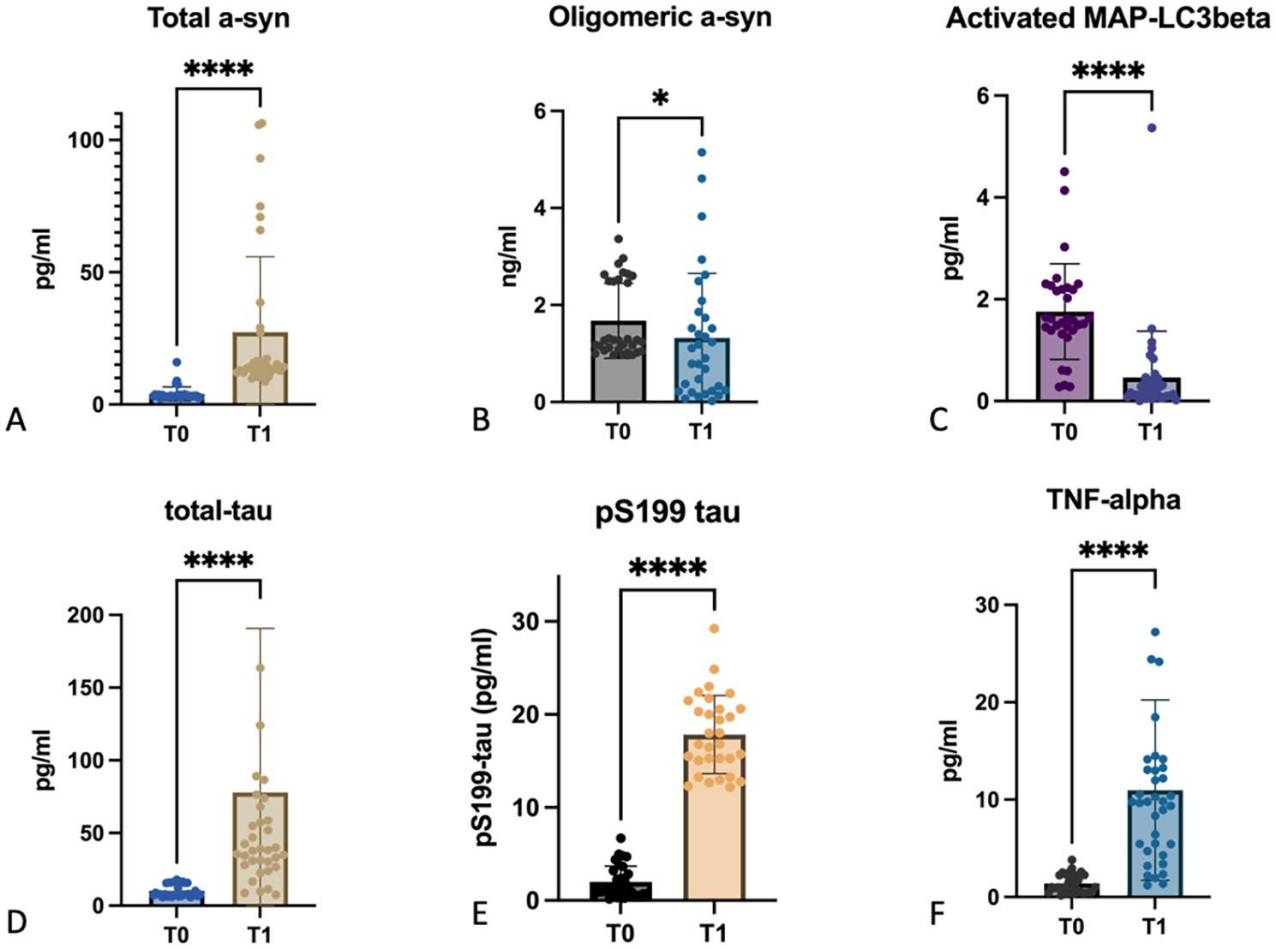
Longitudinal changes in salivary biomarkers in PD patients. Box plots represent the variation in salivary concentration of key molecular biomarkers from early stage (T0) to 4‐year follow‐up (T1) in PD patients. Statistical significance was assessed using Wilcoxon signed‐rank tests; *p < 0.01; ****p < 0.0001.

A significant increase in total a‐syn concentration was observed (*Z* = −4.918, *p* < 0.0001), as shown in Figure 1A. The median salivary concentration of total a‐syn increased from 3.28 pg/mL (IQR 2.9–3.7) at T0 to 14.24 pg/mL (IQR 12–27) at T1. The concentration of oligomeric a‐syn significantly decreased over time (*Z* = −2.618, *p* < 0.01) (Figure 1B), with a median value of 1.3 ng/mL (IQR 1.1–2.5) at T0, which dropped to 0.9 ng/mL (IQR 0.3–1) at T1. Similarly, salivary total tau and pS199tau levels showed a significant increase from T0 to T1 (*Z* = −4.862, *p* < 0.0001; *Z* = −4.782, *p* < 0.001 respectively) (Figure 1D,E). The median total tau concentration was 7.860 pg/mL (IQR 7–15) at T0 and increased to 38.62 pg/mL (IQR 29–79) at T1, while the median pS199tau concentration raised from 1.8 pg/mL (IQR 0.7–2.8) to 17.4 pg/mL (IQR 15–20). Conversely, the concentration of activated MAP‐LC3b—indicative of autophagolysosomal activity—significantly decreased from T0 to T1 (*Z* = −4.413, *p* < 0.0001) (Figure 1C), with median values dropping from 1.601 pg/mL (IQR 1.3–2.2) to 0.2149 pg/mL (IQR 0.09–0.4). Finally, TNFa levels in saliva exhibited a marked increase (*Z* = −4.937, *p* < 0.0001), as illustrated in Figure 1F. Median salivary TNFa concentration rose from 1.041 pg/mL at T0 to 9.769 pg/mL at T1.

### 3.4 Changes in Clinical Severity of Patients From T0 to T1

A significant clinical progression was observed in both motor and non‐motor domains from T0 to T1. In detail, a significant worsening was detected in the following clinical scales: H&Y stage (*Z* = −5.160, *p* < 0.001); UPDRS III (*Z* = −5.164, *p* < 0.0001); NMSS (*Z* = −5.161, *p* < 0.0001); MoCA (*Z* = −4.699, *p* < 0.001). Values are reported in Table 1.

**TABLE 1:** Demographic and clinical data of participants.

Subjects	Age	Sex	Disease duration	H&Y, T0	T1	UPDRS III, T0	T1	UPDRS IV, T1	NMSS, T0	T1	MoCA, T0	T1
PD	68 (62–74)	23% female	5 (4.5–6)	1 (1–2)	2 (2–2.5)	14 (11–20)	26 (20–33.5)	4 (0–12)	20 (13–31)	46 (26–66)	28 (27–29)	26 (25–28)
HC	67 (63–73)	22% female										

*Note:* Values are expressed as median (IQR).

Abbreviations: H&Y, Hoehn and Yahr scale; MoCA, Montreal Cognitive Assessment; NMSS, Non‐Motor Symptoms Scale; PD, Parkinson's disease; UPDRS, Unified Parkinson's Disease Rating Scale.

### 3.5 Correlations Between Salivary Biomarkers and Clinical Scales

At T0, a significant positive correlation was observed between salivary p‐tau levels and NMSS scores (*r* = 0.441, *p* = 0.011). Additionally, a significant positive correlation was detected between MAPLC3b and pS199tau levels (*r* = 0.518, *p* = 0.002), whereas a strong negative correlation emerged between TNFa and p‐tau levels (*r* = −0.736, *p* < 0.001).

**FIGURE 2:**
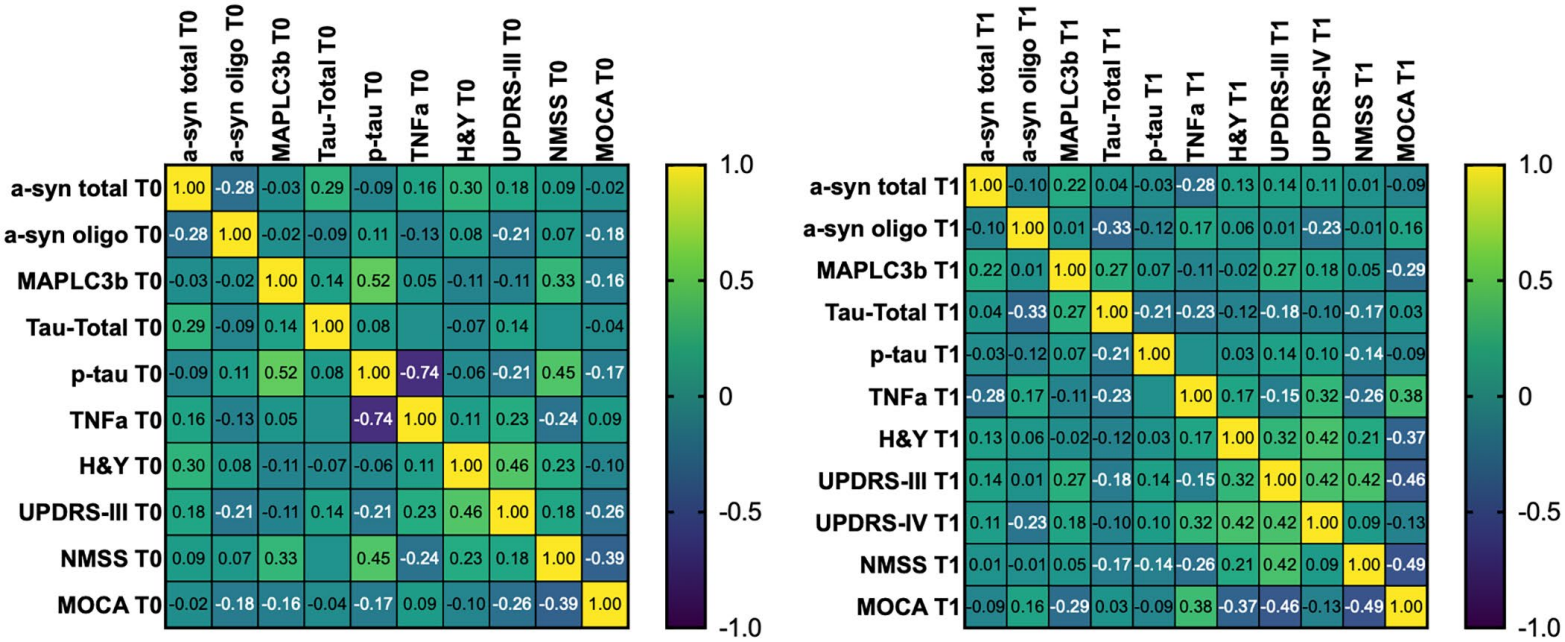
Spearman correlation matrices at baseline (T0) and follow‐up (T1). Heatmaps display Spearman correlation coefficients between salivary biomarkers and clinical variables at T0 (panel A) and T1 (panel B). Warmer colors indicate stronger positive correlations, while cooler colors represent negative correlations. In addition to correlations between biomarkers and clinical scales, the matrices also include inter‐biomarker correlations. Only correlation coefficients are reported in the matrix cells. Significant associations are highlighted and discussed in the main text.

At T1, a significant negative correlation was found between MAPLC3b levels and MoCA scores (*r* = −0.288, *p* = 0.040), while TNFa levels showed a significant positive correlation with both UPDRS IV (*r* = 0.321, *p* = 0.032) and MoCA scores (*r* = 0.380, *p* = 0.014). Additionally, oligomeric a‐syn levels were significantly correlated with total tau levels (*p* = 0.023). The correlation data is shown in the correlation matrix shown in Figure 2.

### 3.6 Predictive Value of Salivary Biomarkers at T0 for Clinical Worsening

**FIGURE 3:**
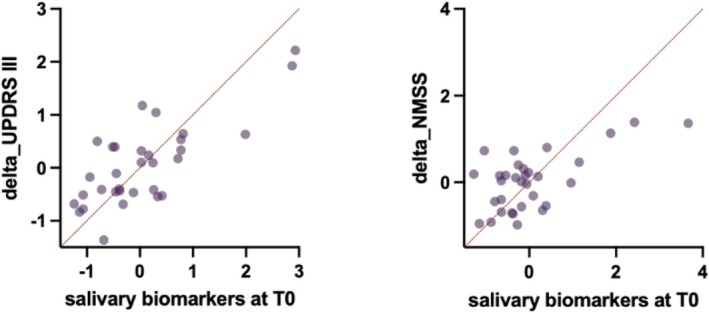
Multiple linear regression analyses using salivary biomarkers at baseline (T0) to predict clinical progression. The left panel shows the results of a multiple linear regression model evaluating the predictive value of *Z*‐scored salivary biomarkers at T0 on motor symptom progression, expressed as delta_UPDRS III (change in UPDRS III score from T0 to T1). The right panel illustrates the corresponding model for non‐motor symptom progression, measured as delta_NMSS (change in Non‐Motor Symptoms Scale total score from T0 to T1). All salivary biomarkers were standardized as *Z*‐scores prior to analysis to ensure comparability across variables. Included biomarkers were total a‐syn, oligomeric a‐syn, total tau, phosphorylated tau (pS199‐tau), MAP‐LC3b, and TNFa. The *y*‐axes represent clinical worsening (delta scores), while the *x*‐axes reflect the combined predictive contribution of baseline salivary biomarkers. Significant predictors are detailed in the main text.

In the multiple linear regression analyses, salivary total a‐syn (*p* < 0.001; *B* = 0.561; SE = 0.136), oligomeric a‐syn (*p* = 0.004; *B* = 0.421; SE = 0.134), total tau (*p* = 0.010; *B* = −0.384; SE = 0.137), and pS199tau (*p* = 0.034; *B* = 0.566; SE = 0.252) measured at T0 emerged as significant predictors of motor symptom progression, as measured by delta_UPDRS III. Furthermore, TNFa levels at T0 significantly predicted the progression of non‐motor symptoms (delta_NMSS) (*p* = 0.028; *B* = −0.682; SE = 0.292) (Figure 3).

### 3.7 Accuracy of Alpha Synuclein at T1 in Discriminating Patients From Controls

**FIGURE 4:**
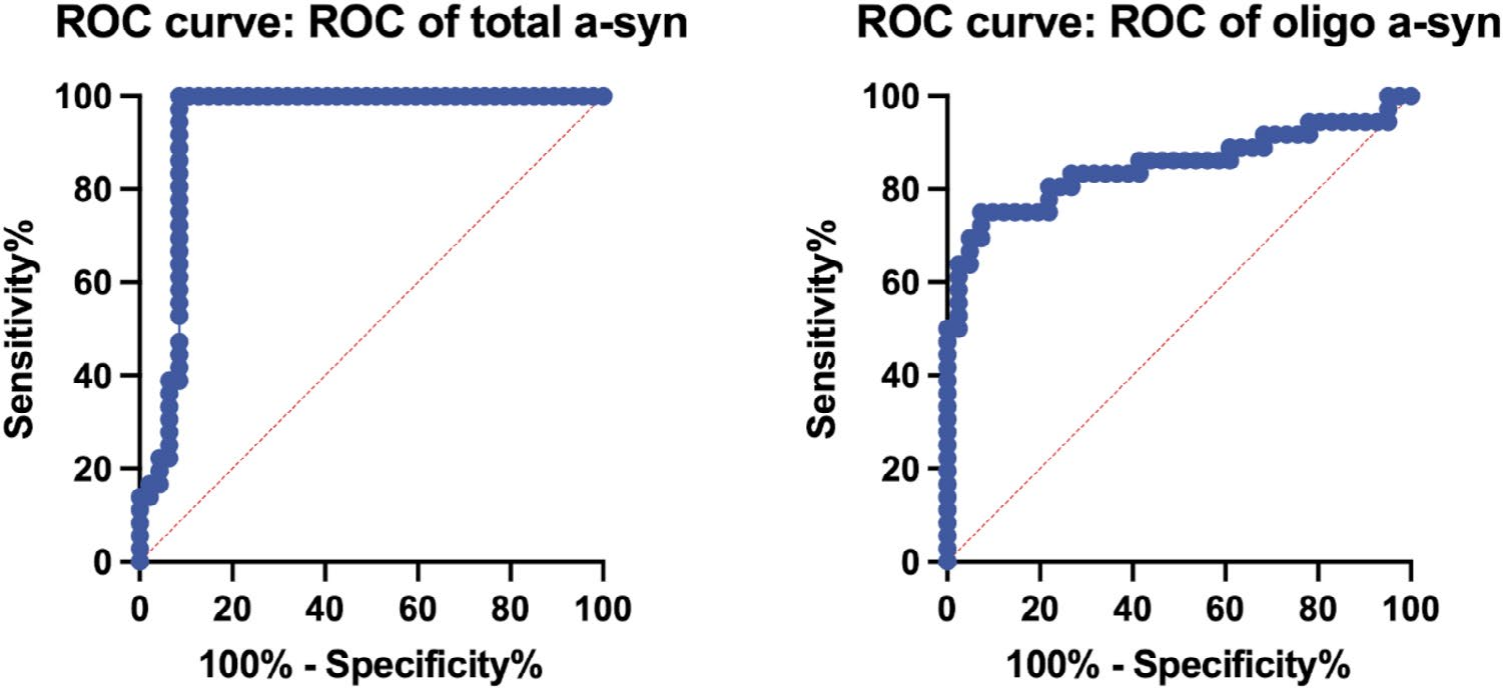
Diagnostic performance of salivary α‐synuclein species in distinguishing PD patients from healthy controls. Receiver operating characteristic (ROC) curves for salivary total α‐synuclein (left) and oligomeric α‐synuclein (right) measured at 4‐year follow‐up (T1).

The diagnostic accuracy of oligomeric α‐synuclein was 85% (AUC = 0.85), with a sensitivity of 75%, specificity of 93%, and a Youden index of 67.68 at the optimal cut‐off threshold (> 0.326). In comparison, total α‐synuclein showed an accuracy of 93% (AUC = 0.93), with a sensitivity of 100%, specificity of 91.49%, and a Youden index of 91.49 at the optimal threshold (> 8.64). See Figure 4.

## 4 Discussion

In the present study, we provide a salivary biomarkers 4‐year follow‐up, highlighting their significant variation in comparison to disease onset, in which de novo PD patients had been molecularly assessed for the first time. We also evaluated how the concentration of salivary biomarkers measured at time T0 could be predictive of the clinical progression.

We observed a significant change in all biomarkers under investigation from time T0 to time T1. In detail, we observed a significant increase in salivary concentration of total a‐syn, pS199‐tau, total tau, and TNFa. Conversely, salivary levels of activated MAPLC3b and oligomeric a‐syn were drastically reduced at time T1 compared to time T0. Progression of the disease was observed for motor and non‐motor features. We did not observe a significant correlation between oligomeric and total a‐syn and clinical score at T1, as well as at T0. Conversely, we found significant correlations of MAPLC3b, pS199‐tau, and TNFa salivary levels with motor and non‐motor scores. Moreover, we found that oligomeric a‐syn and total a‐syn, as well as tau species (both total and pS199‐tau) at disease onset, were able to predict progression of motor symptoms, while TNFa salivary levels were able to predict non‐motor symptoms progression.

### 4.1 Alpha‐Synuclein

In the PD patients investigated at T1, after a 4‐year follow‐up from T0, we found an increase in total salivary a‐syn. This could be due to a release of soluble monomers from the surface of intracellular a‐syn fibrils or from the overexpression of a‐syn in salivary secreting cells and in the autonomic fibers by which they are innervated. The dramatic reduction of oligomeric a‐syn we found at T1 is in line with previous studies in CSF, in which an increase in soluble oligomeric a‐syn species marks the early stages of the disease [23, 24, 25], while in the subsequent phases, the formation of intracellular fibrillar aggregates reduces their release in biological fluids [26]. Moreover, changes in the molecular conformation of a‐syn oligomers in the different stages of the disease may affect the antigenic structure that might not be recognized by conventional ELISA‐based antibody assays, necessitating alternative detection methods. Our findings are consistent with those reported in our previous studies conducted on independent cohorts of PD patients with varying disease duration and severity. Indeed, in our previous study on a cohort of 60 PD patients with a disease duration of 6.7 ± 10.5 years and an H&Y of 1.8 ± 2.1, we detected salivary values of total a‐syn and oligomeric a‐syn comparable to those observed at T1 in the present cohort [27]. These values were also confirmed in a further cohort of 112 PD patients with a disease duration of 6.29 ± 5.03 years and an H&Y of 2.11 ± 0.74 [28], comparable to the patients enrolled in the present follow‐up study.

### 4.2 Tau Species

A significant increase in total and pS199tau levels was observed with disease progression. Tau concentrations in biological fluids have been associated with axonal damage in numerous neurodegenerative diseases, including PD. It is likely that in the more advanced stages of the disease there is a greater axonal injury at the autonomic nerve fibers innervating the salivary glands and therefore a greater concentration of total tau delivered in saliva [29].

### 4.3 MAPLC3 Beta

The observed reduction in salivary activated MAP‐LC3b levels during the more advanced stages of the disease is presumably attributable to the accumulation of intracellular α‐synuclein aggregates, which inhibit autophagolysosome formation by downregulating intracellular autophagic pathways [30]. Indeed, it has been demonstrated that in the advanced stages of synucleinopathies, a dysfunction of the autophagic pathways occurs, contributing to the neuropathological progression of the disease [31, 32].

### 4.4 TNF Alpha

TNFa levels significantly increased from T0 to T1. Similarly to altered autophagy, it has been demonstrated that the progressive formation of intracellular a‐syn aggregates can induce macrophages and microglia to acquire a pro‐inflammatory phenotype with increased production of proinflammatory cytokines such as TNFa, IL‐6, and IL‐1 [30, 33]. We can speculate, therefore, that aggregates of a‐syn in peripheral tissues and peripheral fibers, such as the autonomic fibers innervating salivary glands, can exert this effect on inflammatory cells such as macrophages, leading to an increased salivary concentration of TNFa.

### 4.5 Correlations Between Salivary Biomarkers and Clinical Scales

Correlations findings offer insights into the complex interplay between salivary biomarkers and clinical features in PD, particularly regarding non‐motor symptoms, cognitive domain, and underlying pathophysiological mechanisms. At baseline (T0), the positive correlation between salivary pS199‐tau levels and NMSS scores suggests that tau pathology is possibly linked to the severity of non‐motor symptoms. This aligns with accumulating evidence implicating tau aggregation in the more malignant PD phenotypes [34]. The strong positive correlation at T0 between MAPLC3b and p‐tau levels reinforces the hypothesis of a pathogenic loop wherein autophagy dysfunction may facilitate tau accumulation, potentially exacerbating neurodegenerative processes. Interestingly, the marked inverse relationship between TNFa and pS199‐tau levels at T0 could reflect a compensatory anti‐inflammatory response or distinct temporal dynamics between inflammatory activation and tau pathology during disease progression [35]. At follow‐up (T1), the significant association between salivary oligomeric a‐syn and total tau levels supports the concept of tau/a‐syn co‐pathology, which may synergistically contribute to neurodegeneration and clinical progression [36]. Furthermore, the negative correlation at T1 between MAPLC3b and MoCA scores suggests that autophagy dysregulation is associated with cognitive worsening, in line with previous reports linking lysosomal dysfunction to PD‐related dementia [37]. Conversely, the positive correlation between TNFa levels and both MoCA and UPDRS IV scores at T1 is difficult to interpret and may require further validation. This finding may reflect a dual role of inflammation—either pathogenic or compensatory—whereby TNFa could be upregulated in response to increasing disease burden.

### 4.6 Predictive Value of Salivary Biomarkers at Baseline for Clinical Worsening

The ability of salivary biomarkers to predict the motor and non‐motor progression of PD is a relevant finding of this study. Indeed, we observed that total and oligomeric a‐syn and total and pS199‐tau at T0 were able to predict progression of motor symptoms. This data is in line with recent studies in CSF, showing the potential of a‐syn seeding aggregation assay in correlating with disease severity and predicting disease progression [38]. It is possible that high concentrations of a‐syn oligomers at the onset correlate with an increased spreading of a‐syn pathology to different neuronal territories with a consequent severe clinical progression [39]. In support of this, we observed that the kinetic parameters obtained via Real‐Time Quaking Induced Conversion (RT‐QuIC) in the saliva of de novo PD patients correlate with clinical severity, indicating that an increased salivary seeding‐competent a‐syn oligomers are responsible for a worsened clinical phenotype [13]. Concerning the sign of the association in our multiple linear regression model, with higher baseline total tau levels negatively associated with motor symptom worsening over 4 years (i.e., greater total tau at T0 predicted a smaller increase in delta‐UPDRS III scores), it could appear counter‐intuitive. This finding may reflect a differential role or dynamic balance between total and phosphorylated tau species. Indeed, one possible explanation is that, as pathological tau phosphorylation progresses, a shift occurs from detectable total tau to phosphorylated forms, which may reduce the measurable pool of total tau in peripheral fluids such as saliva, as a consequence of the progressive extensive intracellular aggregation of tau, with reduced extracellular release. Lastly, regarding the prediction of non‐motor symptom progression, we found a significant predictive value only for TNFa levels, suggesting that increased inflammatory activity at the early stages of the disease may contribute to a widespread worsening of PD clinical features, as reported also by transcriptomics analyses [40].

### 4.7 Accuracy of Oligo and Total a‐Syn With Disease Progression

Finally, we confirmed the sustained diagnostic accuracy of oligomeric a‐syn even 4 years after diagnosis. Although levels of oligomeric a‐syn decreased with disease progression, its diagnostic performance remained robust, with an accuracy of 85%, compared to the 99% reported at early disease stages [14]. In contrast, total α‐synuclein at T1 demonstrated excellent accuracy in distinguishing PD from HC (98%), markedly higher than the 55% observed in our previous study [14], likely reflecting the progressive increase in total α‐synuclein levels with disease progression.

### 4.8 Limitations and Strengths

A key limitation of this study is the reduced number of patients who completed the follow‐up: only 43 out of the original 80 patients from the previously described de novo PD cohort [13] were re‐evaluated after 4 years. This reduction was largely due to the overlap of the follow‐up period with the COVID‐19 pandemic, which affected patient retention and in‐person assessments. Nevertheless, despite the limited sample size, the consistency of biomarker dynamics, the strength of associations, and the significant predictive results suggest a robust trajectory that warrants replication in larger cohorts. Moreover, at T0, we did not observe any significant differences in clinical scores or biomarker levels between patients who completed the follow‐up and those who dropped out, suggesting that attrition is unlikely to have biased our data interpretation, except for the MoCA score, which was slightly higher in those who completed the follow‐up. However, even in the dropout group, the MoCA remained within the normal range, suggesting that this difference is unlikely to have biased our data interpretation. Lastly, regarding day‐to‐day variability of biomarker levels, although some degree of intraindividual variability is expected, a previous work has shown that under standardized conditions, this variability is relatively low for salivary a‐syn levels [16]. Nonetheless, this remains a potential source of noise and should be further characterized in dedicated methodological studies.

Importantly, this is the first longitudinal, multi‐pathway biomarker study conducted on salivary samples in PD. It demonstrates the feasibility and utility of using a simple, noninvasive biological matrix such as saliva, in combination with reproducible and accessible ELISA methodologies, to predict and track disease progression. These findings pave the way for future studies in larger and multicenter cohorts aimed at validating the prognostic value of salivary biomarkers and establishing their clinical applicability for routine monitoring and stratification of PD patients.

## 5 Conclusions

In conclusion, our findings support the potential utility of salivary biomarkers in predicting clinical progression of PD. We observed dynamic changes in multiple biomolecular pathways—α‐syn, tau, autophagy, and inflammation—over a 4‐year period, some of which showed predictive value for motor and non‐motor symptom worsening. However, these results need further validation in larger, multicenter cohorts with additional time points to confirm the reproducibility and clinical relevance of our preliminary findings.

## Author Contributions

Maria Ilenia De Bartolo, Daniele Belvisi, Giovanni Fabbrini, Alfredo Berardelli, and Giorgio Vivacqua contributed to the conception and design of the study. Maria Ilenia De Bartolo, Matteo Costanzo, Carolina Cutrona, Massimo Marano, Romina Mancinelli, and Giorgio Leodori contributed to the acquisition of data. Maria Ilenia De Bartolo, Claudia Caturano, Francesco Emanuele Bellomi, and Giorgio Vivacqua contributed to the ELISA measurements and the analysis of data. Maria Ilenia De Bartolo, Daniele Belvisi, and Giorgio Vivacqua conducted final statistical analysis and data interpretation. Maria Ilenia De Bartolo, Daniele Belvisi, Alfredo Berardelli, and Giorgio Vivacqua contributed to drafting the manuscript.

## Conflicts of Interest

The authors declare no conflicts of interest.

## Data Availability

The data that support the findings of this study are available from the corresponding author upon reasonable request.
